# Endocrine disruptors and children’s health

**DOI:** 10.55730/1300-0144.6129

**Published:** 2025-11-06

**Authors:** Ersen KARAKILIÇ, Durmuş DOĞAN, Emre Sedar SAYGILI

**Affiliations:** 1 Division of Endocrinology, Department of Internal Medicine, Faculty of Medicine, Çanakkale Onsekiz Mart University, Çanakkale, Turkiye; 2 Division of Pediatric Endocrinology, Department of Pediatric Medicine, Faculty of Medicine, Çanakkale Onsekiz Mart University, Çanakkale, Turkiye

**Keywords:** Endocrine disruptors, child health, obesity, child development disorders, puberty, thyroid

## Abstract

Endocrine-disrupting chemicals (EDCs) are environmental contaminants that disrupt hormonal regulation by mimicking, inhibiting, or modifying endocrine signaling pathways. EDCs are commonly present in plastics, pesticides, industrial byproducts, and personal care products and pose substantial health risks, particularly to vulnerable groups such as infants and children. Early-life exposure is especially concerning due to the developing detoxification systems, the immaturity of the blood–brain barrier, and the ongoing organ differentiation, making these periods highly susceptible to EDCs’ harmful effects. Moreover, exposure during critical developmental periods, such as sex differentiation and neurodevelopment, can lead to significant long-term developmental impairments that persist into later life.

Perinatal and childhood exposure to EDCs has been linked to various adverse health outcomes, including neurodevelopmental delays, impairments in reproductive health, obesity, type 2 diabetes, thyroid dysfunction, and even a heightened risk of certain malignancies. These effects are mediated through various mechanisms, including direct modulation of hormone receptors, disruption of genetic regulation, and interference with endocrine feedback systems. Alterations in endocrine signaling, particularly disruptions in thyroid hormone homeostasis, may also indirectly impair cognitive development, increasing the risk of attention disorders and intellectual impairment.

Although regulatory measures to reduce EDC exposure are crucial, current restrictions remain insufficient. Moreover, as new EDCs emerge, ongoing research is essential to understand their risks and develop effective strategies to minimize their potential harm. Protecting future generations requires a proactive approach that combines public health awareness, strong regulations, and ongoing scientific research. This review highlights the potential risks of EDCs exposure in children and highlights the significance of multidisciplinary research and policy efforts.

## 1. Introduction

Endocrine-disrupting chemicals (EDCs) are environmental contaminants that disrupt hormonal homeostasis, adversely affecting biological systems. These compounds can affect the endocrine system by mimicking natural hormones or disrupting hormonal balance [1]. Thousands of natural and synthetic chemicals, widely found in plastics, pesticides, industrial chemicals, and personal care products, act as EDCs. These compounds disrupt hormonal signaling and contribute to reproductive dysfunction, neurodevelopmental delays, metabolic disorders, and malignancies [2,3]. Children and fetuses are especially vulnerable to EDC exposure due to their heightened vulnerability during key developmental stages. Exposure during these sensitive periods can interfere with organ system formation and differentiation, resulting in long-term health consequences [4–6]. Thus, a comprehensive understanding of EDCs’ mechanisms and impacts is essential for developing effective protective strategies for children and future generations.

EDCs disrupt the endocrine system through multiple mechanisms, including direct receptor binding, mimicking or blocking natural hormone activity, altering hormone synthesis and metabolism, and influencing gene expression [7]. EDCs can activate or inhibit estrogen, androgen, and thyroid hormone receptors and modulate other nuclear receptors, including glucocorticoid, mineralocorticoid, and aryl hydrocarbon receptors, thereby disrupting endocrine signaling [8]. Additionally, epigenetic mechanisms regulated by hormones during early life are considered fundamental mediators of the long-term effects of EDCs exposure [9]. Mechanisms like deoxyribonucleic acid (DNA) methylation, histone modifications, and micro ribonucleic acid (microRNA) expression can mediate the transgenerational effects of prenatal exposure. They can disrupt cellular functions on their own or synergistically with one another, a phenomenon known as the “cocktail effect.” Nevertheless, the molecular pathways responsible for this synergistic effect remain incompletely understood and require further investigation [10]. Consequently, early-life exposure to EDCs has been linked to a heightened risk of childhood diseases, including neurodevelopmental disorders, reproductive dysfunction, obesity, type 2 diabetes, thyroid dysfunction, and even a heightened risk of certain malignancies [8,11].

## 2. Pathways of exposure to endocrine disruptors

EDCs enter the human body mainly via ingestion, inhalation, and skin absorption. The majority of EDCs exhibit lipophilicity, which enables their accumulation in adipose tissue and contributes to a prolonged half-life [12]. Persistent EDCs, such as dichlorodiphenyltrichloroethane (DDT), can persist in the human body for years. However, nonlipophilic chemicals have shorter half-lives and can be rapidly excreted [13]. Nevertheless, continuous exposure to nonpersistent EDCs, such as bisphenol A (BPA), through daily-use products like plastic containers and thermal paper receipts can sustain their presence in the body, leading to cumulative effects over time [13].

Inhalation and ingestion are associated with significant absorption levels, often correlating with higher systemic concentrations of EDCs. Moreover, ingestion is often the primary route of EDC exposure, primarily through dietary intake. These chemicals are commonly found in food packaging materials, pesticides, and other food-related products, contributing to widespread contamination of the food supply [12]. For instance, BPA and phthalates from plastic containers and linings can leach into food, especially when heated, making them a significant source of human exposure [10,14].

Indoor air exposure to airborne EDCs and contact with consumer products such as textiles, cleaning agents, and cosmetics constitute significant human exposure pathways. EDCs are inhaled when volatile and semivolatile organic compounds from building materials, furniture, and household products are released into the air [15,16].

Dermal absorption of lipid-soluble chemicals is a significant pathway for exposure to various substances in personal care products. The formulation of these products plays a crucial role in influencing the extent and efficiency of dermal absorption [17,18]. Notably, chemicals such as bisphenols and triclosan, commonly present in cosmetics, can penetrate the skin and contribute to systemic exposure [17].

Breast milk serves as a significant route of exposure to EDCs in infants, raising concerns due to potential health risks associated with these compounds [19]. The mammary gland in lactating mothers has a high affinity for lipid-soluble substances, leading to the bioaccumulation and subsequent transfer of EDCs through breast milk [20]. Due to its high lipid content, breast milk represents a significant exposure pathway for lipophilic EDCs, which tend to accumulate in adipose tissue and persist in the body. The concentration of EDCs in breast milk varies depending on geographical location, environmental exposures, and maternal lifestyle factors [13]. This exposure route is particularly concerning during critical developmental windows, as early-life exposure to EDCs can disrupt endocrine regulation, neurodevelopment, and metabolic programming, potentially leading to long-term health consequences in children.

## 3. Developmental vulnerabilities of infants and children

Infants and children have higher exposure to EDCs and greater biological sensitivity than adults [21]. These differences arise from variations in feeding habits, behaviors, physiological characteristics, and toxicokinetics unique to early life stages. For instance, infants consume more water and food relative to their body weight, exhibit higher respiration and absorption rates, and are frequently exposed to environmental toxins through hand-to-mouth behaviors [6,11,13]. Furthermore, their thinner skin structure and higher surface area-to-volume ratio increase dermal absorption of lipid-soluble toxins. At the same time, the immaturity of the blood–brain barrier heightens the risk of neurological damage.

In addition, fetuses and infants have low levels of cytochrome P450 enzymes, which metabolize environmental toxins. Consequently, these chemicals can persist in circulation for extended periods and accumulate at higher tissue concentrations [22,23]. EDCs can disrupt critical time-dependent and synchronized biological processes during development, leading to epigenetic changes that may heighten the risk of neurodevelopmental disorders, cardiometabolic diseases, and sexual developmental disorders later in life [13]. Breast milk serves as a significant route of exposure to persistent EDCs, with studies showing that breastfed infants may exhibit even higher serum concentrations of certain EDCs than their mothers [21]. Consequently, due to their unique and developing physiology, infants and children have higher exposure to EDCs and a diminished ability to eliminate these compounds, making them particularly susceptible to their harmful effects [24].

## 4. Infant growth

Exposure to EDCs during critical developmental periods, such as pregnancy and infancy, occurs alongside the development of the organ systems, substantially increasing the risk of lifelong adverse health effects in affected individuals. Furthermore, perinatal exposure to EDCs can disrupt key processes involved in growth, development, and hormonal regulation in infants, leading to profound consequences for their overall health trajectory. These disruptions not only affect health during infancy and childhood but also increase the susceptibility to chronic disorders in adulthood, such as metabolic syndrome, diabetes, obesity, and neurodevelopmental disorders [6].

Prenatal exposure to phthalates has been linked to lower prepubertal body weight and alterations in sex-specific social behaviors [25]. Similarly, prenatal BPA exposure can alter the expression of estrogen-responsive genes in the developing brain, potentially impairing neurodevelopment and behavior [26]. Moreover, BPA has been shown to alter DNA methylation patterns in genes associated with obesity, potentially increasing the risk of obesity in adulthood [8].

Fetal exposure to EDCs can also negatively impact birth weight. For example, phthalate exposure has been shown to impair placental functions during the prenatal period, restricting nutrient and oxygen transfer to the fetus. This disruption is associated with low birth weight and intrauterine growth restriction [27]. Similarly, BPA disrupts fetal insulin and leptin signaling, leading to metabolic imbalances and reduced birth weight [28]. These effects may also contribute to rapid postnatal weight gain, excessive fat accumulation, and an elevated risk of cardiometabolic disorders in later life [29].

Tributyltin has been implicated in triggering inflammatory responses contributing to reduced birth weight [30]. Additionally, polychlorinated biphenyls (PCBs) and dioxins can alter maternal thyroid hormone levels, negatively affecting fetal brain development and growth [31]. Furthermore, prenatal exposure to mixtures of organochlorine compounds and metals has been linked to decreased birth weight, highlighting the compounded risks posed by combined chemical exposures [32].

Prenatal exposure to EDCs, including metals, phthalates, and BPA, has also been linked to an increased risk of preterm birth [33]. These chemicals interfere with placental function, disrupt hormonal regulation, and alter inflammatory responses, all of which are essential for sustaining a healthy pregnancy.

## 5. Neurodevelopment

Brain development is a complex process that begins during the embryonic period and continues through puberty. During these sensitive windows, the effects of endogenous hormones and the neuroendocrine system on brain development are critical. Steroid hormones, thyroid hormones, and hypothalamic peptides play key roles in synaptic formation, dendritic outgrowth, and the maturation of neuronal connections. Thyroid hormones’ crucial role in neurodevelopment has been demonstrated in many human and animal models [13,34].

EDCs pose a significant threat to neurodevelopment during critical developmental periods due to their ability to cross the placental and blood–brain barriers [6,13]. Prenatal and early childhood exposure to EDCs has been associated with long-term issues such as neurodevelopmental disorders, behavioral problems, cognitive deficits, and psychiatric conditions. EDCs also disrupt multiple endocrine pathways (thyroid, glucocorticoid, insulin, and estrogen), leading to alterations in neuronal organization, gene expression, and neurotransmitter balance with lifelong consequences [13,35]. For example, prenatal and postnatal exposure to polybrominated diphenyl ethers (PBDEs) has been linked to reduced attention span and lower social skills in four-year-old children [36]. Additionally, early prenatal exposure to suspected EDC mixtures has been associated with lower cognitive function levels at age seven, particularly among boys.

The neurotoxic effects of EDCs depend on the type of chemical, duration of exposure, and dosage. These effects can manifest through various mechanisms. For example, substances like BPA exert estrogen-like activities that adversely impact brain development [37]. BPA disrupts synaptic plasticity by altering neurotransmitter levels, including GABA and glutamate [38]. Studies in rats have demonstrated that BPA exposure affects critical brain regions, including the prefrontal cortex, hippocampus, and hypothalamus [39]. A study examined the neurodevelopmental effects of prenatal and early childhood exposure to BPA and nonylphenol (NP) in mother–child pairs. The findings revealed that these chemicals have distinct influences on brain development in early childhood [40]. In particular, urinary NP concentrations in boys aged two to three years showed a negative correlation with intelligence quotient (IQ) and verbal comprehension index scores. Similarly, BPA exposure in girls was linked to changes in motor scores and Wechsler Preschool and Primary Scale of Intelligence – Fourth Edition processing speed scores.

The central role of thyroid hormones in neurodevelopment underscores the serious consequences that can result when EDCs disrupt them. Disruptions in thyroid function caused by several EDCs have been linked to behavioral issues, attention deficits, and impaired neuronal connectivity. These effects contribute to conditions such as attention deficit hyperactivity disorder (ADHD), intellectual disabilities, and learning difficulties [41,42]. The role of thyroid dysfunction in these mechanisms will be discussed in more detail in the following section.

Pesticides also significantly impact neurodevelopment. Organophosphate pesticides can disrupt synaptic transmission by inhibiting acetylcholinesterase, leading to social behavior disorders [13,43]. For instance, prenatal exposure to these substances can cause lasting impairments in children’s cognitive development [44].

## 6. Thyroid

Thyroid hormones are essential for neurodevelopment, growth, energy metabolism, and the immune system. The hypothalamic–pituitary–thyroid axis is highly sensitive to disruptions, and even slight variations in maternal or fetal hormones can impair offspring development, particularly neurodevelopment.

A growing body of evidence indicates that EDC exposure can harm children’s health by interfering with thyroid hormone function. Phthalates, for instance, may disrupt thyroid hormone balance, leading to neuroendocrine dysfunction and increasing the risk of behavioral disorders, including ADHD [10]. Similarly, PCBs interfere with thyroid hormone levels, impairing neuronal connections and resulting in intellectual disabilities, social behavior problems, and attention deficits. Furthermore, hippocampal development impairments and learning difficulties have also been identified in individuals exposed to PCBs [21].

The harmful effects of EDCs on thyroid hormone levels may arise through multiple mechanisms. For instance, perchlorate inhibits the iodide transporter, a critical component of thyroid hormone biosynthesis[45]. Beyond this, disruptions can occur at various levels of thyroid regulation, including hormone synthesis, transport, metabolism, and receptor function [46]. BPA, genistein, and PCBs have been found to disrupt thyroid hormone transport proteins, leading to alterations in the balance between free and bound circulating thyroid hormones [47]. Per- and polyfluoroalkyl substances (PFAS) have also been associated with abnormal thyroid function in newborns and children by interfering with various stages of thyroid hormone synthesis and potentially influencing thyroid autoimmunity [48,49].

These disruptions in thyroid hormone regulation may also influence hormone levels in pregnant women and potentially impact fetal development. A study in pregnant women found that higher BPA levels during early pregnancy were associated with lower total thyroxine (T4) levels and a reduced free T4 to free triiodothyronine (T3) ratio [41]. Similarly, research on women of reproductive age has shown that exposure to PBDEs has been associated with an increased risk of hypothyroidism in this population [50]. Moreover, several human studies have reported a correlation between elevated urinary phthalate levels and increased thyroid-stimulating hormone (TSH) levels, further supporting the role of EDCs in thyroid dysfunction [46]. Additionally, emerging hypotheses propose that prolonged exposure to EDCs may even act as a potential trigger for thyroid cancer, highlighting the broader implications of endocrine disruption [11].

These effects have also been investigated in more extensive cohort studies. A study has demonstrated that exposure to PCBs during pregnancy leads to elevated free T4 levels in both mothers and newborns [51]. Additionally, prenatal exposure to high levels of perchlorate has been linked to cognitive deficits in children, likely due to disruptions in thyroid hormone regulation. This exposure has been associated with lower IQ scores by age three, and notably, levothyroxine treatment did not alleviate these adverse effects [45]. A comprehensive Norwegian cohort study concluded that higher prenatal exposure to phthalates was associated with altered thyroid hormones and an increased risk of developing ADHD [52]. Additionally, a cohort study measuring urinary BPA levels and serum thyroid hormone levels in pregnant women found that BPA exposure during pregnancy was associated with lower total T4 levels in mothers. It reduced TSH levels in male neonates [53].

While most scientific research has focused on prenatal and early-life exposure to pollutants, some studies have also explored their effects on children and young adults. A study conducted in Brazil analyzed the concentrations of metals—including chromium, manganese, mercury, and lead—in children’s blood, and EDC levels detected in their hair. The findings revealed that elevated exposure to these substances was associated with decreased free thyroid hormone levels [54].

Assessing the relationship between thyroid dysfunction and pollutant exposure in epidemiological studies presents significant challenges due to the heterogeneity of populations, pollutant types, and their complex interactions. These challenges are even more pronounced when evaluating the effects of such exposures on fetal development, particularly fetal brain development. Ongoing research continues to advance our understanding of this issue. The ATHENA project, funded by the European Union, aims to overcome the limitations of current testing methods by developing innovative analytical strategies to assess better the effects of chemicals that disrupt the thyroid hormone system. As of this writing, the project remains ongoing and has not yet been completed [55].

## 7. Obesity and diabetes mellitus

There is growing evidence that exposure to endocrine disruptors may play a role in the emergence of diabetes and obesity [56]. These effects may arise from maternal exposure during intrauterine life and the child’s exposure to environmental substances.

One key mechanism underlying this association is metabolic programming, wherein intrauterine nutrient deficiency or growth restriction triggers adaptive physiological changes that enhance fetal survival. While these adaptations are beneficial in the short term, they may predispose individuals to obesity and metabolic disorders in adulthood [57]. Therefore, intrauterine growth restriction due to EDC exposure is increasingly recognized as a potential factor contributing to long-term metabolic risk [58].

Beyond their effects on fetal development, EDCs can also directly influence metabolic processes like insulin resistance and beta-cell function, contributing to the pathogenesis of metabolic syndrome, obesity, and diabetes. Notably, numerous studies have demonstrated that BPA and phthalates can trigger these metabolic disorders [59]. BPA is a selective estrogen receptor modulator and has been implicated in accelerating adipogenesis and postnatal somatic growth. Epidemiologic studies in both adults and children suggest an association between high urinary BPA concentrations and an increased risk of obesity and diabetes [60,61]. Similarly, certain cohort studies have demonstrated an association between prenatal phthalate exposure and childhood obesity [62,63]. PFAS are also recognized as obesogenic chemicals, with birth cohort studies linking their exposure to decreased neonatal weight and an elevated likelihood of childhood obesity [64]. Notably, a metaanalysis found that perfluorooctanoic Acid (PFOA) exposure in children was associated with a 25% increase in body mass index [65]. Although longer-chain PFAS, such as PFOA and perfluorooctane sulfonate (PFOS), have been gradually replaced by shorter-chain alternatives, such as perfluorobutanesulfonic acid (PFBS), growing data indicate that these substitutes may also contribute to obesity [66].

Growing evidence indicates that early-life exposure to PCBs, BPA, dioxins, and perfluorinated compounds may impact the immune system, potentially elevating the risk of immune-related disorders, including type 1 diabetes [11]. However, establishing precise cause-and-effect relationships in these cases remains challenging due to confounding factors and limitations in exposure assessment, and many more prospective studies should be conducted to shed light on them.

## 8. Male reproductive system

Many EDCs exhibit estrogenic, antiandrogenic, and steroidogenesis-disrupting properties, and fetal testes are highly sensitive to these EDCs’ effects. Exposure during this critical developmental period has been linked to the testicular dysgenesis syndrome, a condition characterized by abnormalities in reproductive system development, impaired semen function, and an increased risk of testicular germ cell cancer later in life [67].

During fetal development, testicular descent and male genital formation are regulated by insulin-like growth factor-3 produced by Leydig cells. Endocrine disruptors can interfere with this pathway, possibly contributing to congenital abnormalities such as hypospadias, cryptorchidism, and oligospermia [68–70]. Notably, exposure to phthalates has been associated with an increased risk of genital abnormalities, including hypospadias, reduced penile length, decreased anogenital distance, hormonal imbalances indicative of Leydig cell dysfunction, and reduced testicular volume [71,72]. Furthermore, research indicates that exposure to pesticides may be linked to an increased risk of undescended testicles [73]. Additionally, exposure to DDT has been linked to reduced sperm function and count [74], while BPA has been associated with sperm quality deterioration[75].

## 9. Female reproductive system

Intrauterine exposure to EDCs in females may disrupt ovarian development and contribute to the onset of PCOS (Polycystic Ovary Syndrome), endometriosis, and infertility later in life [76]. Furthermore, exposure to EDCs during early-life stages may disrupt mammary gland development, possibly elevating the risk of breast cancer [77–79].

Studies have shown a potential link between BPA and PFAS exposure and an increased risk of PCOS, an association between phthalates and endometriosis, and a possible relationship between PFAS and endometriosis [2]. BPA is particularly toxic to the ovaries and, beyond its association with PCOS, it also has been linked to vaginal adenosis, cervical sarcoma, uterine polyps, and breast cancer [80].

In response to this growing body of evidence, regulatory measures have been introduced to restrict BPA usage in baby care items, including bottles, pacifiers, food packaging, and toys, to reduce early-life exposure. Even though several EDCs known to affect fertility are no longer manufactured, the emergence of novel EDCs raises concerns that they may exert similarly adverse effects, necessitating ongoing evaluation and regulation [81].

## 10. Puberty

EDCs can significantly affect pubertal development; however, these effects are complex and not fully understood. These chemicals disrupt hormonal function, altering the timing of puberty. For instance, exposure to estrogenic compounds is associated with early puberty, whereas other EDCs can cause delays in pubertal onset [82,83]. The effects of EDCs often exhibit sex-specific variations. In girls, specific EDC exposures have been associated with earlier puberty onset, while others may lead to delays. Similarly, in boys, chemicals such as PCBs have been linked to accelerated puberty, whereas exposure to dioxins and lead has been shown to delay its timing [83,84].

These effects occur primarily by disrupting the hypothalamic–pituitary–gonadal axis, which plays a central role in pubertal development and can act through central or peripheral mechanisms [84,85]. While animal studies provide robust evidence regarding the effects of EDCs on puberty, human studies often yield inconsistent results due to the complexity of environmental exposures and genetic factors [86,87]. Additionally, prenatal and postnatal exposure to EDCs can have different effects, though the precise windows of vulnerability are not yet fully understood [88]. The impact of EDCs on puberty varies based on the type, timing, and dosage of exposure, emphasizing the need for further research to gain deeper insights into these complex interactions.

## 11. Strategies for reducing exposure to endocrine disruptors in children

EDCs pose significant risks to children’s health, particularly during the fetal, neonatal, and pubertal stages, making it essential to minimize exposure during these critical periods. Lifestyle modifications, policy regulations, and awareness-raising initiatives are essential strategies to reduce EDC exposure in children. Plastic use, especially products containing BPA, should be minimized in favor of safer alternatives such as glass or stainless steel. Adopting an organic diet can reduce exposure to pesticides and agricultural chemicals, while using natural cleaning products and avoiding canned foods can help minimize household sources of EDCs [13,88,89].

The European Union has classified EDCs as high-risk substances and prohibited their use in pesticides. Similarly, comprehensive international regulatory measures are recommended to mitigate their harmful effects. Additionally, awareness programs for mothers before conception could help reduce EDC exposure during critical fetal and neonatal periods, minimizing potential health risks. Therefore, education programs that raise parental awareness and enhance knowledge about EDCs are urgently needed to mitigate their potential health risks. Combining lifestyle changes, policies, and educational efforts is pivotal in reducing EDC exposure and protecting children’s long-term health.

## 12. Conclusion

Following the surge in industrialization in the 20th century and the rise in consumption, numerous new chemicals have entered our daily lives. Unfortunately, over time, many of these chemicals have been found to possess endocrine-disrupting properties and are associated with various health disorders. These substances are now known as EDCs.

Infants and children are particularly vulnerable to the harmful effects of EDCs due to their immature defense mechanisms. Moreover, as their organ systems are still developing, early exposure may result in long-term health consequences, including neurological and reproductive disorders, as well as cardiometabolic diseases and even cancer. Moreover, perhaps, in the future, many currently unexplained health issues may be linked to early-life EDC exposure.

A significant challenge in addressing EDCs is identifying these substances. The vast number of potentially harmful chemicals makes it difficult to establish clear cause-and-effect relationships. Even when certain chemicals are identified and banned, their replacements may also pose new risks.

Although research on EDCs is increasing, we are still far from fully understanding their effects. There is a continued need for extensive studies in this area. The effects of EDCs are complex, and their impact evolves. Some health consequences become apparent many years after childhood exposure. Due to many chemicals with unclear effects and the uncertainty of their long-term impacts, there is a critical need for more proactive regulations to protect children. Just as new drugs must undergo rigorous safety testing before reaching the market, chemicals used in products for children should also undergo similar safety assessments before widespread adoption.

EDCs are a growing concern that affects everyone, but children are at the highest risk. To protect future generations, we must advance comprehensive research and implement proactive and stringent regulations without delay.
